# Biotransformation of Natural Products and Phytochemicals: Metabolites, Their Preparation, and Properties

**DOI:** 10.3390/ijms24098030

**Published:** 2023-04-28

**Authors:** Kateřina Valentová

**Affiliations:** Laboratory of Biotransformation, Institute of Microbiology of the Czech Academy of Sciences, Vídeňská 1083, 14220 Prague, Czech Republic; kata.valentova@email.cz

The term “biotransformation” refers to the process by which various compounds are biocatalyzed and enzymatically modified, as well as the metabolic changes that occur in organisms as a result of exposure to xenobiotics. This Special Issue focuses on both these connotations of biotransformation and specifically on the biotransformations of natural products and phytochemicals.

Metabolites of xenobiotics may have different biological and biophysical properties compared to their parent compounds, which can result in inactivation, toxication, or alteration in activity. In contrast, phytochemicals and other natural products are gaining increasing attention for their significant benefits in preventing and managing chronic diseases. However, these compounds often suffer from low bioavailability and extensive metabolism, with their metabolites being responsible for their biological effects [1,2,3,4,5].

Therefore, it is essential to prepare and study the metabolites of phytochemicals in detail. To tackle the complexity of these compounds, enzymatic methods have recently been introduced in the preparation of their (tentative) metabolites. This Special Issue aimed to cover a range of topics, including the discovery of new chemical and biochemical methods for metabolite preparation (including multienzyme methods), advanced structural and analytical methods for metabolite characterization, and the determination of the biological and biophysical properties of metabolites.

An example of a study of the biotransformation of natural products in plant tissue is the investigation of the ability of *Silybum marianum* (L.) Gaertn (milk thistle, Asteraceae) leaves to convert phenolic acids into silymarin flavonolignans. The study found that expanding leaves had the full capacity to convert di-caffeoylquinic acid to a silymarin complex. Only medium-sized leaves had the metabolic capacity to convert callus components into silymarin, indicating that cotyledon-derived callus can efficiently produce compounds that can be bioconverted to flavonolignans in the leaf tissue of *S. marianum* [6]. Another important example is the bioconversion of polyunsaturated fatty acids, such as linoleic and arachidonic acids, which, together with their various metabolites, are involved in several biological processes. They influence cell membrane fluidity and permeability, modulate platelet activity and coagulation, regulate lymphocyte activity and inflammation, preserve the permeability of the glomerular barrier, influence podocyte physiology, and play a role in renal fibrosis with implications for biological and clinical manifestations of idiopathic nephrotic syndrome [7].

The biotransformations of licochalcones and xanthohumol (5) by the fungi *Mucor hiemalis* and *Absidia coerulea* resulted in one new glucosylated metabolite and four new and three known sulfated metabolites. Licochalcone A 4′-sulfate showed less cytotoxic activity against human melanoma (A375P), breast adenocarcinoma (MCF-7), and lung carcinoma (A549) cell lines compared to its parent licochalcone A, with IC_50_ values in the range of 27–43 [8]. In contrast, the biotransformations of licoisoflavanone, glycyrrhisoflavone, echinatin, and isobavachalcone by *Aspergillus niger* strain KCCM 60332 afforded twelve new compounds by a series of reactions including hydroxylation, hydrogenation, epoxidation, hydrolysis, reduction, cyclization, and alkylation. Two compounds exhibited the most considerable cytotoxic activities against all three cell lines investigated, while two others were moderately cytotoxic [9]. The use of microorganisms for biotransformation is a sustainable and eco-friendly alternative to chemical synthesis for repurposing by-products generated by the agri-food industry. *Rhodococcus* strains have been successfully utilized for the biotransformation of bovine and porcine bile acids, including cholic, deoxycholic, hyocholic, and hyodeoxycholic acids. The resulting secosteroids derived from these acids have been found to exhibit potent antifungal activity against several pathogenic fungi such as *Fusarium moniliforme*, *Pleospora betae*, *Pythium ultimum*, *Sarracenia minor*, and *Sclerotinia sclerotiorum*. Compared to their parent acids, these secosteroids demonstrate enhanced efficacy and could prove to be promising agents in the development of novel antifungal therapeutics [10].

Biotransformations with isolated or engineered enzymes are an effective alternative to chemical synthesis in the production of value-added products from natural sources as well. For instance, four glycosyltransferases (GTs) from *Bacillus* sp., including BtGT_16345 from the *B. thuringiensis* GA A07 strain and three GTs (BsGT110, BsUGT398, and BsUGT489) from the *B. subtilis* ATCC 6633 strain were used to biotransform ganoderic acid G (GAG), a saponin from *Ganoderma lucidum*. BsUGT489 and BsGT110 successfully glycosylated GAG into GAG-3-*O*-β-glucoside and GAG-26-*O*-β-glucoside, which showed 54-fold and 97-fold higher aqueous solubilities, respectively, compared to GAG [11]. Enzymatic methods have also been used for the production of tentative metabolites of bioactive compounds. Bacterial arylsulfotransferases such as that derived from *Desulfitobacterium hafniense* have gained popularity in recent years for the sulfation of natural compounds. These enzymes are 3′-phosphoadenosine-5′-phosphosulfate (PAPS)-independent and utilize readily available sulfate donors, such as *p*-nitrophenyl sulfate and phenylsulfate [12,13]. In the case of sulfation of phenolic acids, enzymatic sulfation of monohydroxyphenolic acids failed, probably due to enzyme inhibition. In contrast, the same reaction was successful for dihydroxyphenolic acids (3,4-dihydroxyphenylacetic acid and 3,4-dihydroxyphenylpropionic); the target products were obtained as mixtures of the 3′- and 4′-sulfates. In contrast to chemical sulfation, the 3′-sulfates were the major products of enzymatic sulfation [14].

In summary, the papers featured in this Special Issue demonstrate the significance of biotransformation as a vital area of research, particularly in the conversion of natural bioactive compounds. These papers highlight the successful biotransformation of various compounds, such as phenolic acids into silymarin flavonolignans or sulfated derivatives or polyunsaturated fatty acids, bile acids, chalcones, or saponins into bioactive compounds. The utilization of microorganisms, along with isolated or engineered enzymes, presents a sustainable and environmentally friendly option for producing high-value products from natural sources without the need for chemical synthesis. This research has significant implications for the development of new and innovative approaches to meet the growing demand for eco-friendly and sustainable products.
